# Analysis of influencing factors of passes in the chinese super league

**DOI:** 10.1186/s13102-022-00572-5

**Published:** 2022-10-12

**Authors:** Yue Zeng, Hui Zhang

**Affiliations:** Department of Sport Science, College of Education, Zhejiang University, Hangzhou, China

**Keywords:** Chinese Super League, Mixed linear model, Pass the ball, Influencing factor

## Abstract

**Background:**

In football matches, passing is an important means of organizing attacks, creating shots, changing tactics, and achieving tactical objectives and is a frequently used technique. The purpose of the study was to explore the relevant factors that affect passes in the Chinese Super League (CSL) in different game contexts.

**Methods:**

A total of 1,440 matches (2,880 samples) of 24 teams participating in the CSL in the 2014–2019 seasons were selected as the research object, and a mixed linear model was constructed to analyse the influencing factors of passes.

**Results:**

(a) The passing success rate and the total number of forward passes were greatly affected by the stage of the season; (b) except for the passing success rate in the 30 m attack area, all other pass indicators in the home matches were significantly higher than those in the away matches; (c) the number of passes in the 30 m attack area was mainly affected by the team level (table position quartile); the higher the team level, the greater the number of passes in the 30 m attack area; (d) all passing indices for the matches between the first eight and the last eight were significantly greater than the matches between the first eight; (e) the passing success rate in the 30 m attack area in winning matches was higher than that in drawn matches; (f) the number of forward passes was significantly greater in matches won by two or more goals, and the number of defender passes was significantly greater in losing matches.

**Conclusion:**

The various passing indices of the CSL were affected by different game contexts (including season stage, venue, team level, match type and match outcome), and understanding these influencing factors of passes will help coaches and players better understand football matches.

## Background

In football/soccer matches, passing is an important means of organizing attacks, creating shots, changing tactics, and achieving tactical objectives and is a frequently used technique. At the same time, passing technology is also an important means to connect players in various positions. It can help defenders launch attacks, midfielders organize attacks, and forward players create chances to score goals.

Most current research on passing in football matches focuses on passing networks, personal passing ability, and data analyses. Research on passing networks shows that midfielders often play the most essential role in passing networks, especially offensive midfielders, followed by central defenders [1]. A study revealed that central midfielders were prominent players in the attacking process in the majority of tactical lineups. These players showed the highest levels of connection with their teammates and were significantly relevant in making passes and linking the sectors of the team [2]. Clemente et al. also found that match outcome had a certain relationship with the centrality of the passing network at different positions in the passing sequence [3]. Comparisons of the passing network centrality levels between won and lost matches revealed small increases in degree prestige (inbound pass links) among midfielders and small increases among forwards in matches won. Players who passed more times had higher connection centrality and proximity in the passing network [4].

A study on the passing network of the Chinese Super League (CSL) showed that the network connection, diameter, density, and clustering coefficient of the winning team and the home team were significantly higher than those of the losing team and the away team, while the density and clustering coefficient of the winning team were significantly lower than those of the losing team [5]. An analysis of the passing network characteristics of the teams in the 2014 World Cup showed that there were significant differences in the dependent variables of network density and total links between the teams in the late stage of the match [6].

Passing accuracy is a problem for coaches and players. In particular, the passing direction, power control, and foot touch position are the core factors that affect passing accuracy. Factors to improve passing accuracy include player position, running direction, mutual distance, teammate running speed, and timing [7]. In addition, some studies have analysed changes in the application of players’ passing technique under anaerobic and high-pressure conditions. The more that players are involved in passing training, the greater their internal and external load response. After intermittent anaerobic exercise, the accuracy of the long-pass and short-pass methods decreased significantly. The impact on the accuracy of the long-pass was particularly obvious [8, 9].

The influence of passing data on goals is the focus of technical and tactical analysis in football. Relevant studies have shown a possible correlation between the increase in pass distribution uniformity and goals [10]. The top teams in the season pass more times than other teams. The number of passes in the second half of the match was lower, but the number of goals was higher [11]. A comparison of the passing methods of Spain and South Korea, the champions of the 2010 World Cup in South Africa, found that there were significant differences in the number of short-passes in the first half and the number of short-passes, middle passes, long-passes, flat passes and direct passes in the second half [12]. In the English Premier League, within 5 min before the goal, the passing success rate of the scoring team was significantly higher than the half-time average, while the passing success rate of the losing team was significantly less. However, after the team scored, the number of passes was significantly reduced, and the accuracy of passes was significantly lower than that before the goal [13]. There was no difference in the number of passes between successful and unsuccessful teams in the 2002 World Cup. The only obvious difference was that in the defensive centre (close to the middle line), the losing team tended to have more passes [14]. Liu and Peng used the generalized linear model and data series inference method to analyse the 2014 season of the CSL. The research showed that adding two standard deviations of passing, the passing success rate, and the straight plug could produce increases of 21.6% (± 15.9%), 27.3% (± 17.7%), and 16.9% (± 22.9%) to the team’s probability of winning [15].

Studies have examined the relationship between long- and short-passes and goals. Hughes and Franks analysed the passes leading to goals in the two World Cup finals and found that the long-pass sequence produced more goals than the short-pass sequence [16]. For unsuccessful teams, neither strategy had obvious advantages. Kylie et al. also found that the proportion of goals with longer consecutive passes (five or more passes) was high, with an average of 13 per 1000 passes in Australian A-League football. The proportion of goals with consecutive short-passes (four or fewer passes) was six goals [17].

Recently, a series of studies on the CSL has included indicators related to passing [18–22]. Given the importance of various passing indicators in world cups and professional leagues, this study constructed a mixed linear model to analyse the factors that affect passing in the CSL.

## Methods

The study was approved by the local institutional ethics committee, and we obtained permission for the use of the CHAMPION Sports Information Technology Company.

### Samples

A total of 1,440 matches (2,880 samples) of 24 teams participating in the CSL in the 2014–2019 seasons were selected as the research object (due to the impact of the COVID-19 epidemic, the 2020–2021 seasons of the CSL did not adopt the home-away game system, so its related data are not suitable for this study). During this period, the names of some teams changed (for example, the Chongqing Lifan Club was renamed the Chongqing Dangdai Lifan Club in the 2017 season and the Chongqing Dangdai Lifan SWN Club in the 2018 season). As the basic situation of the team did not fundamentally change, it is still regarded as the same team in the study.

### A mixed linear model of passing

#### Dependent variable

There are many types of passing in football matches. We selected seven indicators as dependent variables for analysis according to previous related studies [23, 24]: the total number of passes, the passing success rate, the number of passes in the 30 m attack area, the passing success rate in the 30 m attack area, the total number of forward passing, the total number of midfield passing and the total number of defender passing (including center back, left back and right back).

#### Fixed effect

The variables (match situations) include the season stage, venue, team level, match type and match outcome. The season stage is divided into the early stage (rounds 1–10), middle stage (rounds 11–20), and late stage (rounds 21–30). The venue is divided into home and away. The team level (according to the rankings after the end of the seasons) is divided into four groups: teams that ranked 1st-4th (CSL) are considered high-level groups, 5th-8th are upper-level groups, 9th-12th are lower-level groups, and 13th-16th are low-level groups. The match type is divided into four categories: the match between the last eight, the match between the last eight and the first eight, the match between the first eight and the last eight, and the match between the first eight. The match outcome is divided into five categories: loss of two or more goals, one goal loss, draw, one goal win and win of two or more goals.

### Random effect

The variable is seasons 2014–2019 of the CSL.

#### Mixed linear model of the passing index

According to the purpose of the study, the mixed linear models of each passing index are established as follows (taking the total number of passes as an example):

Y_pass_=β_0_ + β_1_Stage_ij_ + β_2_Venue_ij_  + β_3_Level_ij_  + β_4_Type_ij_  + β_5_Outcome_ij_ + T_ij_ + Ɛ_ij_

The above formula is a mixed linear model of passing. Y_pass_ is the dependent variable, that is, all kinds of passing indicators. β_0_ is the intercept, β_1_Stage_ij_, β_2_Venue_ij_, β_3_Level_ij_, β_4_Type_ij_, and β_5_Outcome_ij_ represent the fixed effects and their parameters, such as season stage, venue, team level, match type and match outcome. T_ij_ is a random effect (season), and Ɛ_ij_ is random error. The subscripts i and j are the dependent variable value of the jth observation in the ith sample.

This study chooses the fixed and random effects of the model according to two principles: (1) If the treatment of the explanatory variable that produces the effect is one of the finite treatments of the explanatory variable, and the effect of this treatment on the dependent variable is exactly the one studied is of interest, then this effect is chosen as a fixed effect; (2) if the treatment of the explanatory variable that produces this effect is one of the infinite (usually infinite but also finite) treatments of the explanatory variable, and the treatment can be considered to be randomly selected from a population, this effect is selected as a random effect [25]. Therefore, in the model of this study, the season is used as a random effect, and other variables are used as fixed effects.

#### Model information statistics

Through testing, it was found that the models with a scaled identity covariance structure in the random effects had the best-fitting effect. Table 1 shows the information statistics (AIC and BIC) of models with identity covariance structures (the smaller the value of the information statistics, the better the model fit).

**Table 1:** Information statistics of models with scaled identity covariance structure

Model	AIC	BIC
Total number of passes	33296.319	33308.240
Passing success rate	-8748.241	-8736.320
Number of passes in the 30 m attack area	27788.813	27800.733
Passing success rate in the 30 m attack area	-7319.951	-7308.030
Total number of forward passing	26070.714	26082.634
Total number of midfield passing	31088.731	31100.651
Total number of defender passing	29908.798	29920.718

### Data source and processing

The data came from http://data.champdas.com (the CHAMPION Sports Information Technology Company). Gong et al. verified the accuracy and effectiveness of the Champdas Master Match Analysis System, the Kappa values for the inter-operator reliability were 0.97 and 0.89; the intra-class correlation coefficients and typical errors ranged from 0.90 to 1.00 and ranged from 0.01 to 0.24 for two independent operators within two data collections. [26]. SPSS version 24.0 software (SPSS Inc., Chicago, IL, USA) was used to estimate the mixed linear model. All tests were two-tailed tests, and P < 0.05 was considered statistically significant.

## Results

### Overview of passing data in the Chinese Super League

The CSL had a sample of 480 matches in each season from 2014 to 2019. The sample of each season stage (early stage, middle stage, and late stage) in the six seasons was 960 matches, and the samples of home and away matches were 1440 matches. The samples of each team level group (high level, upper level, lower level and low level) were 720 matches. There were 672 matches between the last eight, 768 matches between the last eight and the first eight, 768 matches between the first eight and the last eight, and 672 matches between the first eight. There were 510 matches with a loss of two or more goals, 558 matches with a one-goal loss, 744 matches with a draw, 588 matches with a one-goal win, and 510 matches with a win of two or more goals. Table 2 shows the descriptive statistical values of the dependent variables of each passing index in the CSL.

**Table 2:** Descriptive statistics of the CSL in the 2014–2019 seasons

Variable	$\overline {\rm{X}} \pm {\rm{Sd}}$	95%CI
Total number of passes	385.784 ± 95.153	382.307, 389.260
Passing success rate (%)	76.456 ± 6.100	76.233, 76.679
Number of passes in the 30 m attack area	91.652 ± 35.595	90.352, 92.953
Passing success rate in the 30 m attack area (%)	67.673 ± 7.308	67.406, 67.940
Total number of forward passing	58.504 ± 29.007	57.444, 59.564
Total number of midfield passing	177.714 ± 69.041	175.191, 180.236
Total number of defender passing	137.265 ± 56.614	135.197, 139.334

### Total number of passes

Table 3 shows the fixed effect and random effect analysis of the total number of passes and the passing success rate in the CSL. Among them, the match venue, match type and match outcome had a very significant impact on the total number of passes. The total number of passes in the home matches was 33.284 units higher than that in the away matches (P < 0.001), and the total number of passes in the matches between the last eight and the matches between the first eight and last eight was 44.964 (P = 0.015) and 57.664 (P < 0.001) units higher than that in the match between the first eight. Compared with the drawn matches, the total number of passes in the matches with a one-goal win was 21.113 (P < 0.001) units less, but the total number of passes in the matches with a one-goal loss and a loss of two or more goals was 19.709 (P < 0.001) and 29.603 (P < 0.001) units more, respectively.

**Table 3:** Analysis of the influencing factors of the total number of passes and the passing success rate

	Total number of passes					Passing success rate			
	*β*	*SE*	*t*	*P*		*β*	*SE*	*t*	*P*
*Fixed effect parameter*									
Intercept	354.188	10.681	33.159	< 0.001		0.748	0.008	92.305	< 0.001
Early stage	3.787	3.538	1.070	0.285		-0.006	0.002	-2.660	0.008
Middle stage	-6.676	3.530	-1.891	0.059		0.011	0.002	4.665	< 0.001
Late stage	0^#^	0				0^#^	0		
Home	33.284	2.975	11.188	< 0.001		0.012	0.002	6.193	< 0.001
Away	0^#^	0				0^#^	0		
Low-level group	-27.103	20.939	-1.294	0.198		-0.013	0.016	-0.782	0.436
Lower-level group	-30.046	20.007	-1.502	0.136		-0.010	0.015	-0.642	0.523
Upper-level group	-14.830	10.392	-1.427	0.157		-0.005	0.008	-0.629	0.531
High-level group	0^#^	0				0^#^	0		
Between the last eight	44.964	18.149	2.477	0.015		0.160	0.014	1.156	0.251
Last eight vs. first eight	0.523	18.101	0.029	0.977		-0.002	0.014	-0.156	0.877
First eight vs. last eight	57.664	4.173	13.817	< 0.001		0.024	0.003	9.038	< 0.001
Between the first eight	0^#^	0				0^#^	0		
Loss of two or more goals	29.603	4.655	6.359	< 0.001		0.017	0.003	5.753	< 0.001
One goal loss	19.709	4.412	4.467	< 0.001		0.005	0.003	1.588	0.112
One goal win	-21.113	4.430	-4.766	< 0.001		-0.005	0.003	-1.729	0.084
Win of two or more goals	-7.767	4.707	-1.650	0.099		0.009	0.003	2.802	0.005
Draw	0^#^	0				0^#^	0		
*Covariance parameter*									
Residual	5975.105	160.229	37.291 (Wald z)	< 0.001		0.003	6.716E-5	37.249 (Wald z)	< 0.001
Season	678.307	118.746	5.712 (Wald z)	< 0.001		0.0004	7.294E-5	5.873 (Wald z)	< 0.001

Note: # it is parameter redundancy, so it is set to zero (the same below)

The impact of the season stage and the team level on the total number of passes was not significant (all P > 0.05). Table 3 also shows that the difference in the total number of passes of different seasons was very significant [β = 678.307, z = 5.712, P < 0.001].

### The passing success rate

The impact of each dimension’s season stage on the passing success rate was very significant (Table 3). The passing success rate in the early stage was 0.006 (P = 0.008) units less than that in the late stage, but the passing success rate in the middle stage was 0.011 (P < 0.001) units higher than that in the late stage. In addition, the match venue, the match type and the match outcome had a significant impact on the passing success rate. The passing success rate in the home matches was 0.012 units higher than that in the away matches (P < 0.001), and the passing success rate in the matches between the first eight and last eight was 0.024 units higher than that in the matches between the first eight (P < 0.001). From the perspective of the match outcome, whether the matches had a loss of two or more goals or a win of two or more goals, their passing success rates were higher than those of the drawn matches, 0.017 (P < 0.001) and 0.009 (P = 0.005) units, respectively.

There were no significant effects on the passing success rate of teams at different levels (all P > 0.05). However, the difference in the passing success rate in different seasons [β = 0.0004, z = 5.873, P < 0.001] was also significant.

### The number of passes in the 30 m attack area

Table 4 shows the fixed effect and random effect analysis of the total number of passes and the passing success rate in the 30 m attack area of the CSL. Each dimension in the match venues, the team levels and the match outcomes had a significant effect on the number of passes in the 30 m attack area (high-level group vs. low-level group: P = 0.019, high-level group vs. lower-level group: P = 0.016, other P < 0.001). In addition, the influence of the match between the first eight and last eight on the number of passes in the 30 m attack area was 18.309 units higher than that of the match between the first eight (P < 0.001).

**Table 4:** Analysis of the influencing factors of the number of passes and the passing success rate in the 30 m attack area

	Total number of passes in the 30 m attack area					Passing success rate in the 30 m attack area			
	*β*	*SE*	*t*	*P*		*β*	*SE*	*t*	*P*
*Fixed effect parameter*									
Intercept	87.486	3.721	23.509	< 0.001		0.671	0.008	84.495	< 0.001
Early stage	-0.611	1.358	-0.450	0.653		-6.723E-5	0.003	-0.023	0.82
Middle stage	-1.451	1.355	-1.071	0.284		0.007	0.003	2.293	0.022
Late stage	0^#^	0				0^#^	0		
Home	14.505	1.142	12.706	< 0.001		-0.003	0.002	-1.271	0.204
Away	0^#^	0				0^#^	0		
Low-level group	-17.293	7.236	-2.390	0.019		-0.018	0.015	-1.187	0.238
Lower-level group	-16.847	6.911	-2.438	0.016		-0.008	0.015	-0.556	0.579
Upper-level group	-14.565	3.591	-4.056	< 0.001		-0.012	0.008	-1.624	0.107
High-level group	0^#^	0				0^#^	0		
Between the last eight	14.631	6.295	2.324	0.022		0.013	0.013	0.962	0.338
Last eight vs. first eight	-0.265	6.274	-0.042	0.966		-0.001	0.013	-0.042	0.967
First eight vs. last eight	18.309	1.602	11.432	< 0.001		0.019	0.003	5.349	< 0.001
Between the first eight	0^#^	0				0^#^	0		
Loss of two or more goals	8.297	1.785	4.649	< 0.001		0.004	0.004	0.920	0.358
One goal loss	6.063	1.693	3.582	< 0.001		-0.006	0.004	-1.567	0.117
One goal win	-7.921	1.699	-4.662	< 0.001		0.008	0.004	2.151	0.032
Win of two or more goals	-9.253	1.805	-5.126	< 0.001		0.025	0.004	6.450	< 0.001
Draw	0^#^	0				0^#^	0		
*Covariance parameter*									
Residual	879.937	23.595	37.293 (Wald z)	< 0.001		0.004	0.0001	37.272 (Wald z)	< 0.001
Season	76.118	14.049	5.418 (Wald z)	< 0.001		0.0003	6.468E-5	5.230 (Wald z)	< 0.001

However, the impact of the season stages on the number of passes in the 30 m attack area was not significant (all P > 0.05). Table 4 also shows that the difference in the number of passes in the 30 m attack area in different seasons was very significant [β = 76.118 , z = 5.418, P < 0.001].

### The passing success rate in the 30 m attack area

Table 4 shows that the impact on the passing success rate in the 30 m attack area in the middle stage and in the matches between the first eight and the last eight was 0.007 (P = 0.022) and 0.019 (P < 0.001) units higher than that in the late stage and in the matches between the first eight, respectively. The influence on the passing success rate in the 30 m attack area in matches with a one-goal win and with a win of two or more goals was 0.008 (P = 0.032) and 0.025 (P < 0.001) units higher than in the draw matches, respectively.

However, the venues and the team levels had no significant effect on the passing success rate in the 30 m attack area. The passing success rate in the 30 m attack area in different seasons [β = 0.0003, z = 5.230, P < 0.001] was significantly different.

### The total number of forward passes

Table 5 shows the fixed effect and random effect analysis of the total number of forward, midfield and defender passes in the CSL. The season stage and the venue had a significant effect on the total number of forward passes. The impact of the early stage and the middle stage on the total number of forward passes was 2.374 (P = 0.016) and 5.199 (P < 0.001) units fewer than that in the late stage, respectively. The influence of the home matches on the total number of forward passes was 2.793 units higher than that of the away matches (P < 0.001), which suggests that forwards have more passes in the late stage of the seasons and the home matches. In addition, the impact of the matches between the first eight and the last eight and the matches with a win of two or more goals on the total number of forward passes was 4.281 (P < 0.001) and 2.873 (P = 0.028) units higher than in the match between the first eight and the draw match, respectively.

**Table 5:** Analysis of the influencing factors of the total number of forward, midfield and defender passes

	Total number of forward passes				Total number of midfield passes				Total number of defender passes			
	*β*	*SE*	*t*	*P*	*β*	*SE*	*t*	*P*	*β*	*SE*	*t*	*P*
*Fixed effect parameter*												
Intercept	56.501	5.804	9.734	< 0.001	181.316	11.154	16.256	< 0.001	102.923	10.033	10.258	< 0.001
Early stage	-2.374	0.982	-2.417	0.016	2.534	2.371	1.069	0.285	3.712	1.923	1.930	0.054
Middle stage	-5.199	0.980	-5.306	< 0.001	-0.187	2.365	-0.079	0.937	-0.792	1.919	-0.413	0.680
Late stage	0^#^	0	.		0^#^	0			0^#^	0		
Home	2.793	0.826	2.382	0.001	13.020	1.994	6.530	< 0.001	17.023	1.617	10.526	< 0.001
Away	0^#^	0	.		0^#^	0			0^#^	0		
Low-level group	-6.933	11.695	-0.593	0.555	-41.069	22.350	-1.838	0.069	24.524	20.162	1.216	0.227
Lower-level group	-4.934	11.187	-0.441	0.660	-42.914	21.375	-2.008	0.048	21.457	19.283	1.113	0.269
Upper-level group	-1.309	5.806	-0.226	0.822	-34.067	11.096	-3.070	0.003	21.708	10.009	2.169	0.033
High-level group	0^#^	0			0^#^	0			0^#^	0		
Between the last eight	9.965	10.020	0.995	0.323	27.246	19.192	1.420	0.159	6.739	17.293	0.390	0.698
Last eight vs. first eight	5.631	10.013	0.562	0.575	5.819	19.172	0.304	0.762	-12.504	17.278	-0.724	0.471
First eight vs. last eight	4.281	1.159	3.694	< 0.001	29.914	2.797	10.695	< 0.001	23.147	2.269	10.203	< 0.001
Between the first eight	0^#^	0	.		0^#^	0			0^#^	0		
Loss of two or more goals	1.081	1.295	0.835	0.404	14.087	3.126	4.506	< 0.001	13.783	2.537	5.434	< 0.001
One goal loss	1.388	1.227	1.131	0.258	6.489	2.961	2.192	0.028	10.893	2.402	4.535	< 0.001
One goal win	-0.179	1.232	-0.145	0.884	-9.671	2.973	-3.253	0.001	-11.124	2.412	-4.612	< 0.001
Win of two or more goals	2.873	1.309	2.194	0.028	-0.917	3.161	-0.290	0.772	-11.422	2.564	-4.454	< 0.001
Draw	0^#^	0			0^#^	0			0^#^	0		
*Covariance parameter*												
Residual	460.098	12.359	37.478 (Wald z)	< 0.001	2683.208	72.049	37.241 (Wald z)	< 0.001	1765.367	47.403	37.241 (Wald z)	< 0.001
Season	253.829	39.687	6.396 (Wald z)	< 0.001	896.520	143.696	6.239 (Wald z)	< 0.001	742.222	117.178	6.334 (Wald z)	< 0.001

There was no significant difference between each dimension of match type and the total number of forward passes (all P > 0.05). There were significant differences in the total number of forward passes in different seasons [β = 253.829, z = 6.396, P < 0.001].

### The total number of midfield passes

Table 5 shows that the impact of the venue, the team level, the match type and the match outcome had a significant difference in the total number of midfield passes. Among them, the influence of the home matches on the total number of midfield passes was 13.020 units higher than that of the away matches (P < 0.001), the impact of the lower-level group and the upper-level group was 42.914 (P = 0.048) and 34.067 (P = 0.003) units lower than that of the high-level group, respectively, and the impact in the matches between the first eight and the last eight was 29.914 units higher than in the matches between the first eight (P < 0.001). In addition, the different match outcomes had a greater impact on the total number of midfield passes. The matches with a loss of two or more goals and a loss of one goal were 14.087 (P < 0.001) and 6.489 (P = 0.028) units higher than the matches with a draw, and the matches with a one-goal win were 9.671 units lower than the matches with a draw (P = 0.001).

There was no significant difference in the total number of midfield passes between season stages (all P > 0.05). There were significant differences in the total number of midfield passes in different seasons [β = 896.520, z = 6.239, P < 0.001].

### The total number of defender passes

The impact of the home match, the upper-level group and the match between the first eight and the last eight on the total number of defender passes was 17.023 (P < 0.01), 21.708 (P = 0.033) and 23.147 (P < 0.01) units higher than that of the away matches, the high-level group and the match between the first eight, respectively. The impact of each dimension’s match outcomes on the total number of defender passes was very significant (Table 5). The total number of defender passes in the matches with a loss of two or more goals and a one-goal loss was 13.783 and 10.893 units higher than in the matches with a draw, respectively, and the total number of defender passes in matches with a one-goal win and a win of two or more goals was 11.124 and 11.422 units lower than in the matches with a draw, respectively (all P < 0.001).

There was no significant difference in the total number of defender passes between season stages (all P > 0.05). There were significant differences in the total number of defender passes in different seasons [β = 742.222, z = 6.334, P < 0.001].

## Discussion

### The impact of the season stage

We investigated whether various passing indices change with the progress of the season. To our knowledge, this study is the first to investigate the impact of the three stages of the CSL on passes. We found that the passing success rate and the total number of forward passes were greatly affected by the season stage. The passing success rate was highest in the middle of the season. The reason may be that by the middle of the season, the players of each team have become more skilled at passing the ball, their tactical playing methods have gradually matured and become fixed, and they have maintained good physical fitness so that the passing success rate is at a high stage. The total number of forward passes is greater in the late stage of the season, which may be related to each team’s desire for more goals or more shots to obtain a better league ranking (Table 6).

**Table 6:** Goal, shot and shot on target during 2014–2019 seasons (N = 960)

	Early stage	Middle stage	Late stage	F	P	${\text{E}}_{\text{R}}^{2}$
Goal	1.347 ± 1.207^B^	1.503 ± 1.270^Aa^	1.543 ± 1.272^Aa^	6.587	0.001	0.005
Shot on target	4.312 ± 2.498^Bb^	4.351 ± 2.481^Bb^	4.624 ± 2.519^Aa^	4.450	0.012	0.003
Shot	12.263 ± 5.070	12.622 ± 4.951	12.726 ± 4.775	2.333	0.097	0.002

Note: 1) Note: In each row of Table 6, those with different uppercase letters showed very significant differences between groups (P < 0.01). Those with different lowercase letters showed significant differences between groups (P < 0.05). Those with the same lowercase letter showed no significant differences between groups (P > 0.05) [27]

### The impact of the venue

There is no doubt that home has many advantages over away. The first is the size of the field. The length and width of each football field are not fixed but within the fluctuation range of a fixed value. This is very important for some teams that mainly control the ball. The second is the fans. The home players can often integrate the support of the fans into their power. For teams fighting away from home, the formation of fans is undoubtedly a kind of pressure. The third is turf. The softness and length of the turf are different. This will affect the rolling speed of the ball after contacting the grass, including the speed of the players running on the turf. The turf of the stadium is trimmed and maintained according to the requirements of the home team. Home advantage has been examined by many previous studies in football [28–30]. Carmichael and Thomas observed significantly more passes in home matches than in away matches in the English Premier League [31]. Our results further support this conclusion: except for the passing success rate in the 30 m attack area, all other pass indicators in the home matches were significantly higher than those in the away matches. This result is also in accordance with Chen et al.’s research [32].

### The impact of the team level

The team level has a greater impact on the number of passes in the 30 m attack area in the CSL. The better the team’s rankings are, the greater the number of passes in the 30 m attack area. However, the effect of different team levels on the passing success rate of the 30 m attack area was not significant. This result is consistent with another study. Yang et al. reported that the number of entry passes in the final 1/3 of the field (final 1/3 entries) in the CSL was 46.20$\pm$16.1 for the top-ranked 1–4 group, 44.39±13.1 for the upper-middle-ranked 5–8 group, 43.76$\pm$13.6 for the lower-middle-ranked 9–12 group, and 39.34$\pm$12.6 for the lower-ranked 13–16 group. The differences were significant (P = 0.032) [19].

### The impact of the match type

When teams play against different opponents, different tactics are often used, so some studies have examined the match type as a variable [24][33]. We found that all passing indices for the matches between the first eight and the last eight were significantly greater than the matches between the first eight. This result is in accordance with a previous analysis that found that the top teams made more passes and scored more goals, while teams relegated to a lower level had, on average, a lower number of passes and goals [11]. However, there are also some studies showing that passing indices have a smaller contribution to ranking points obtained in a professional game [34] and are less predictable when considering team quality and home advantage [35, 36].

### The impact of match outcomes

Many match analyses divide the outcomes of matches into wins, draws and losses. This study further subdivides outcomes into five categories and finds that the total number of passes and the number of passes in the 30 m attack area in the drawn matches were lower than in losing matches and higher than in winning matches. However, the passing success rate and the passing success rate in the 30 m attack area in winning matches were higher than those in drawn matches, especially in matches won by two or more goals. The main reason for this is that defender passing and midfield passing in losing matches were significantly greater than in drawn matches. However, the number of forward passes in the matches won by two or more goals was significantly higher. The results of this study are basically consistent with those of Cao et al., who examined 187 non-drawn matches in the 2019 Chinese Super League [24]. The number of passes in the 30 m attack area of the winning teams was 91.75 ± 31.18 and that of the losing teams was 96.35 ± 36.01, but the difference was not significant (P = 0.188). In contrast, the passing success rate in the 30 m attack area of winning teams was 69.84 ± 7.15 and that of the losing teams was 67.75 ± 7.07, and the difference was significant (P = 0.005).

### Limitations

Passing is the most common game behaviour in football matches, and there are many indicators related to passing. Due to the limitation of data sources, our research did not analyse other passing indicators (through ball, key pass, cross, long-pass, short-pass, cross-pass, etc.) for an in-depth and detailed analysis. In addition, the level of different leagues varies greatly, which also affects passing. Therefore, a broader analysis can lead to better conclusions about the role of passing in the game and the factors that influence it.

## Conclusion

The mixed linear analysis model of passing based on different match situations can help us further understand the passing behaviour of the Chinese Super League. The results showed that (a) the passing success rate and the total number of forward passes were greatly affected by the season stage; (b) except for the passing success rate in the 30 m attack area, all other pass indicators in the home matches were significantly higher than those in the away matches; (c) the number of passes in the 30 m attack area was mainly affected by the team’s level; the higher the team’s level, the greater the number of passes in the 30 m attack area; (d) all passing indices for the matches between the first eight and the last eight were significantly greater than the matches between the first eight; (e) the passing success rate in the 30 m attack area in winning matches was higher than in the drawn matches, especially in the matches with a win of two or more goals; (f) the number of forward passes was significantly greater in the matches won by two or more goals, and the number of defender passes was significantly greater in the losing matches.

## Data Availability

The datasets used and/or analysed during the current study are available from the corresponding author on reasonable request.

## Abbreviations

CSL: Chinese Super League.
AIC: Akaike information criterion.
BIC: Bayesian information criterion.
